# Utility of the refined EBMT diagnostic and severity criteria 2023 for sinusoidal obstruction syndrome/veno-occlusive disease

**DOI:** 10.1038/s41409-024-02215-4

**Published:** 2024-01-29

**Authors:** Hiroya Ichikawa, Kimikazu Yakushijin, Keiji Kurata, Takahiro Tsuji, Naoko Takemoto, Miki Joyce, Yuri Okazoe, Ruri Takahashi, Sakuya Matsumoto, Rina Sakai, Akihito Kitao, Yoshiharu Miyata, Yasuyuki Saito, Shinichiro Kawamoto, Katsuya Yamamoto, Mitsuhiro Ito, Tohru Murayama, Hiroshi Matsuoka, Hironobu Minami

**Affiliations:** 1 Division of Medical Oncology and Hematology, Department of Medicine, Kobe University Hospital and Graduate School of Medicine, Kobe, Japan; 2 Department of Oncology and Hematology, Hyogo Prefectural Harima-Himeji General Medical Center, Himeji, Japan; 3 Department of Artificial Intelligence and Digital Health Science, Kobe University Graduate School of Medicine, Kobe, Japan; 4 Division of Molecular and Cellular Signaling, Kobe University Graduate School of Medicine, Kobe, Japan; 5 Transfusion Medicine and Cell Therapy, Kobe University Hospital and Graduate School of Medicine, Kobe, Japan; 6 Laboratory of Hematology, Division of Medical Biophysics, Kobe University Graduate School of Health Sciences, Kobe, Japan; 7 Department of Hematology, Hyogo Cancer Center, Akashi, Japan; 8 Department of Integrated Analyses of Bioresource and Health Care, Kobe University Graduate School of Medicine, Kobe, Japan

**Keywords:** Medical research, Signs and symptoms

## Abstract

Sinusoidal obstruction syndrome/veno-occlusive disease (SOS/VOD) is a life-threatening complication of hematopoietic stem cell transplantation (HSCT). Early diagnosis of SOS/VOD is associated with improved clinical outcomes. In 2023, the refined European Society for Blood and Marrow Transplantation diagnostic and severity criteria (refined EBMT criteria 2023) have been advocated. The revision has introduced new diagnostic categories, namely; probable, clinical, and proven SOS/VOD. In addition, the Sequential Organ Failure Assessment (SOFA) score has been newly incorporated into the SOS/VOD severity grading. We performed a retrospective analysis to evaluate the utility of these criteria. We analyzed 161 cases who underwent allogeneic HSCT. We identified 53 probable, 23 clinical, and 4 proven SOS/VOD cases. Probable SOS/VOD was diagnosed a median of 5.0 days earlier (interquartile range: 2–13 days, *P* < 0.001) than that of clinical SOS/VOD. The development of probable SOS/VOD alone was associated with a significantly inferior survival proportion compared to non-SOS/VOD (100-day survival, 86.2% vs. 94.3%, *P* = 0.012). The SOFA score contributed to the prediction of prognosis. Consequently, the refined EBMT criteria 2023 demonstrated the utility of SOS/VOD diagnosis and severity grading. Further investigations and improvements in these criteria are warranted.

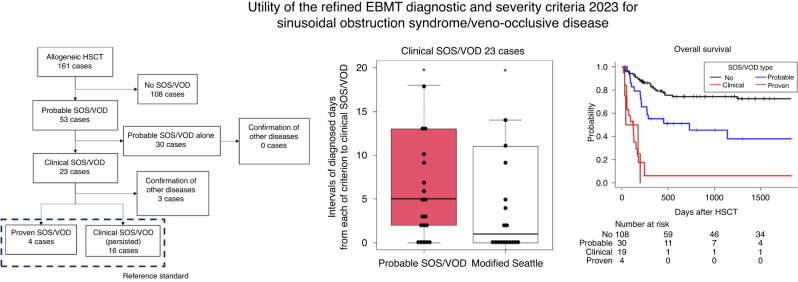

## Introduction

Sinusoidal obstruction syndrome (SOS), also known as veno-occlusive disease (VOD), is a life-threatening complication of hematopoietic stem cell transplantation (HSCT). While the incidence of SOS/VOD is relatively low (approximately 2–15%) among patients undergoing allogeneic HSCT [1–4], particularly, severe SOS/VOD is associated with extremely high mortality [1, 5]. Immediate initiation of SOS/VOD treatment potentially overcomes the poor prognosis [6–8]. Accordingly, the early diagnosis of SOS/VOD is associated with improved clinical outcomes. The attempt to prevent SOS/VOD in adults has not achieved successful results yet [9]. Therefore, the strategy of early diagnosis and treatment initiation has remained to be most important.

Because it was often difficult to perform invasive procedures for histological SOS/VOD diagnosis in the post-HSCT setting, physicians had used the surrogate clinical diagnostic criteria, such as the modified Seattle criteria [10] and Baltimore criteria [11] for SOS/VOD diagnosis. In 2016, the European Society for Blood and Marrow Transplantation (EBMT) advocated the diagnostic criteria for SOS/VOD [12]. Thereafter, these criteria had been generally recognized as gold standards. However, these criteria were challenging for the early detection of SOS/VOD, particularly in the classical SOS/VOD setting (defined as development within 21 days after HSCT) that required serum total bilirubin elevation. In 2020, Cairo et al. proposed other diagnostic criteria, including the parameter of refractory thrombocytopenia [13]. We have reported on the efficacy of these criteria for the early diagnosis of SOS/VOD [14]. Nonetheless, more extensive investigation is lacking.

In 2023, the revision of the EBMT diagnostic and severity criteria (refined EBMT criteria 2023) was advocated [15]. The revision introduced new diagnostic categories, namely; probable, clinical, and proven SOS/VOD. In addition, the Sequential Organ Failure Assessment (SOFA) score [16] was newly incorporated into the SOS/VOD severity grading system. The establishment of “probable SOS/VOD” supposedly enables the earlier diagnosis of SOS/VOD. However, no published data support the validity and efficacy of these refined EBMT criteria 2023. Thus, we performed retrospective analysis to evaluate the utility of these criteria.

## Methods

### Patients

Data of patients aged ≥18 years who underwent allogeneic HSCT at Kobe University Hospital between January 2012 and December 2022 were analyzed retrospectively. We collected and analyzed the clinical data of each patient during admission and visit days, between the beginning of conditioning and the last follow-up. This study was approved by the Ethics Committee of Kobe University Hospital (No. B220141). The requirement for informed consent was waived because of the retrospective nature of the study. The patients who participated in this study were offered the opportunity to opt-out. This study was conducted in accordance with the principles of the Declaration of Helsinki.

### Definitions

Definitions of SOS/VOD based on the modified Seattle criteria and the refined EBMT criteria 2023 are shown in Table 1. In this study, we did not consider the restriction of diagnosed days in the modified Seattle criteria (within 20 days after HSCT). Regarding the refined EBMT criteria 2023, “ultrasound findings suggestive of SOS/VOD” were defined as a positive estimation of HokUS-10 and/or HokUS-6 in this study [17, 18]. In cases where HokUS-10/6 was not performed, the finding of a decrease in the velocity or reversal of portal flow, in addition to the suggestive findings such as hepatomegaly, were considered “suggestive of SOS/VOD”. When we estimated the sensitivity and specificity, clinical SOS/VOD cases, excluding those with histological confirmation of other diseases, were considered as the reference standard.

**Table 1:** Definitions of SOS/VOD upon the modified Seattle criteria and the refined EBMT criteria 2023.

	Refined EBMT criteria 2023		
Modified Seattle criteria	Probable SOS/VOD	Clinical SOS/VOD	Proven SOS/VOD
The presence of 2 or more	The presence of 2 or more	The presence of bilirubin ≥2 mg/dL and	The presence of either
of the following within 20 days	of the following	2 or more of the following	of the following
(1) Bilirubin >2 mg/dL	(1) Bilirubin ≥2 mg/dL	(1) Painful hepatomegaly	(1) Histologically proven
(2) Hepatomegaly	(2) Painful hepatomegaly	(2) Weight gain: >5%	(2) Hemodynamically proven
and/or	(3) Weight gain: >5%	(3) Ascites	(HVPG ≥ 10 mmHg)
Right upper quadrant pain	(4) Ascites		
(3) Weight gain: >2%	(5) Ultrasound and/or elastography		
	suggestive of SOS/VOD		

*SOS/VOD* sinusoidal obstruction syndrome/veno-occlusive disease, *EBMT* European Society for Blood and Marrow Transplantation, *HVPG* hepatic venous pressure gradient.

Definitions of SOS/VOD severity grading based on the refined EBMT criteria 2023, and the SOFA score are shown in Tables S1 and S2. Multiple organ dysfunction (MOD) was defined as the presence of two or more organ dysfunctions that corresponded to each SOFA score ≥2. Exceptionally, thrombocytopenia was taken into account for this assessment only when there was a SOFA score ≥2 increase from the best point [15]. All cases who developed probable SOS/VOD were graded according to the aforementioned methods.

We classified the conditioning regimens as myeloablative upon using any of the following: total body irradiation >8 Gy, intravenous busulfan >7.2 mg/kg, or melphalan >140 mg/m^2^. Other conditioning regimens were classified as reduced-intensity conditioning [19]. Transplantation-related mortality (TRM) was defined as death excluding that caused by primary diseases [20].

### Statistical analysis

Categorical and continuous variables of case characteristics were compared using the Fisher’s exact test and Kruskal–Wallis test, respectively. We compared the intervals of diagnosed days between probable SOS/VOD, the modified Seattle criteria, and clinical SOS/VOD using the Wilcoxon signed-rank sum test. Overall survival was estimated using the Kaplan–Meier method with the log-rank test. Post-hoc tests were conducted using the Bonferroni correction. TRM was described using the cumulative incidence method, considering relapse-related death as a competing risk. Statistical significance was defined as a two-tailed *P* value < 0.05. All statistical analyses were performed using R version 4.1.2 and EZR version 1.55 [21].

## Results

### Patient characteristics

We analyzed a total of 141 patients (corresponding to 161 transplantation cases) who underwent allogeneic HSCT. The patient characteristics are summarized in Table 2. Haploidentical HSCT was not performed in our study cohort. The median follow-up period was 1117 days (range: 33–3942 days) after HSCT in the 97 survivors. Of the 64 patients who died, 15 underwent autopsies. Of the 161 cases, 12 underwent liver biopsies, accordingly with no detection of SOS/VOD. The vast majority of the 161 cases underwent an ultrasound test at baseline, and again when physicians suspected SOS/VOD. This included 12 cases with the HokUS-10/6 estimation method. Ursodeoxycholic acid for SOS/VOD prophylaxis was used in our hospital routinely at the discretion of the physicians. Three patients received defibrotide prophylaxis in the clinical trial [9].

**Table 2:** Case characteristics.

		No SOS/VOD	Probable SOS/VOD	Clinical SOS/VOD	Proven SOS/VOD	Total cases	*P* value
*N*		108	30	19	4	161	
Median (range) age, years		53 (19–69)	49 (19–69)	53 (28–65)	45 (37–55)	52 (19–69)	0.85
Sex (%)	Male	65 (60.2)	17 (56.7)	12 (63.2)	1 (25.0)	95 (59.0)	0.54
	Female	43 (39.8)	13 (43.3)	7 (36.8)	3 (75.0)	66 (41.0)	
Disease (%)	AML	43 (39.8)	10 (33.3)	6 (31.6)	0 (0.0)	59 (36.6)	0.7
	ALL/LBL	24 (22.2)	6 (20.0)	3 (15.8)	1 (25.0)	34 (21.1)	
	MDS	13 (12.0)	3 (10.0)	3 (15.8)	1 (25.0)	20 (12.4)	
	CML/MPN	5 (4.6)	3 (10.0)	1 (5.3)	1 (25.0)	10 (6.2)	
	ML	11 (10.2)	2 (6.7)	1 (5.3)	1 (25.0)	15 (9.3)	
	MM	4 (3.7)	0 (0.0)	0 (0.0)	0 (0.0)	4 (2.5)	
	ATL	3 (2.8)	3 (10.0)	2 (10.5)	0 (0.0)	8 (5.0)	
	AA	2 (1.9)	0 (0.0)	1 (5.3)	0 (0.0)	3 (1.9)	
	Others*	3 (2.8)	3 (10.0)	2 (10.5)	0 (0.0)	8 (5.0)	
Disease status (%)	CR	73 (67.6)	18 (60.0)	5 (26.3)	2 (50.0)	98 (60.9)	0.14
	PR	9 (8.3)	4 (13.3)	3 (15.8)	0 (0.0)	16 (9.9)	
	SD	1 (0.9)	1 (3.3)	0 (0.0)	0 (0.0)	2 (1.2)	
	PD	20 (18.5)	6 (20.0)	8 (42.1)	2 (50.0)	36 (22.4)	
	Unevaluable	5 (4.6)	1 (3.3)	3 (15.8)	0 (0.0)	9 (5.6)	
ECOG PS (%)	0	31 (28.7)	2 (6.7)	3 (15.8)	0 (0.0)	36 (22.4)	<0.01
	1	72 (66.7)	25 (83.3)	9 (47.4)	3 (75.0)	109 (67.7)	
	2	4 (3.7)	2 (6.7)	4 (21.1)	0 (0.0)	10 (6.2)	
	3	0 (0.0)	1 (3.3)	2 (10.5)	1 (25.0)	4 (2.5)	
	4	1 (0.9)	0 (0.0)	1 (5.3)	0 (0.0)	2 (1.2)	
HCT-CI (%)	0	30 (27.8)	10 (33.3)	2 (10.5)	0 (0.0)	42 (26.1)	0.16
	1–2	24 (22.2)	4 (13.3)	7 (36.8)	0 (0.0)	35 (21.7)	
	≥3	54 (50.0)	16 (53.3)	10 (52.6)	4 (100.0)	84 (52.2)	
Source (%)	BM	40 (37.0)	14 (46.7)	10 (52.6)	1 (25.0)	65 (40.4)	0.53
	PBSC	19 (17.6)	4 (13.3)	5 (26.3)	1 (25.0)	29 (18.0)	
	CB	49 (45.4)	12 (40.0)	4 (21.1)	2 (50.0)	67 (41.6)	
Donor (%)	Related	23 (21.3)	2 (6.7)	4 (21.1)	1 (25.0)	30 (18.6)	0.32
	Unrelated	85 (78.7)	28 (93.3)	15 (78.9)	3 (75.0)	131 (81.4)	
Number of transplantation (%)	1	92 (85.2)	24 (80.0)	15 (78.9)	4 (100.0)	135 (83.9)	0.79
	2	14 (13.0)	6 (20.0)	4 (21.1)	0 (0.0)	24 (14.9)	
	3	2 (1.9)	0 (0.0)	0 (0.0)	0 (0.0)	2 (1.2)	
HLA serotype mismatch (%)	No	50 (46.3)	13 (43.3)	10 (52.6)	2 (50.0)	75 (46.6)	0.93
	Yes	58 (53.7)	17 (56.7)	9 (47.4)	2 (50.0)	86 (53.4)	
HLA genotype mismatch (%)	No	45 (41.7)	10 (33.3)	9 (47.4)	2 (50.0)	66 (41.0)	0.75
	Yes	63 (58.3)	20 (66.7)	10 (52.6)	2 (50.0)	95 (59.0)	
Conditioning (%)	MAC	47 (43.5)	10 (33.3)	7 (36.8)	4 (100.0)	68 (42.2)	0.08
	RIC	61 (56.5)	20 (66.7)	12 (63.2)	0 (0.0)	93 (57.8)	
BU containing regimen (%)	No	76 (70.4)	20 (66.7)	9 (47.4)	4 (100.0)	109 (67.7)	0.12
	Yes	32 (29.6)	10 (33.3)	10 (52.6)	0 (0.0)	52 (32.3)	
TBI containing regimen (%)	No	14 (13.0)	2 (6.7)	4 (21.1)	0 (0.0)	20 (12.4)	0.42
	Yes	94 (87.0)	28 (93.3)	15 (78.9)	4 (100.0)	141 (87.6)	
GVHD prophylaxis (%)	TAC + MMF	86 (79.6)	29 (96.7)	15 (78.9)	3 (75.0)	133 (82.6)	0.13
	CyA + MMF	13 (12.0)	1 (3.3)	2 (10.5)	1 (25.0)	17 (10.6)	
	TAC alone	9 (8.3)	0 (0.0)	1 (5.3)	0 (0.0)	10 (6.2)	
	Other	0 (0.0)	0 (0.0)	1 (5.3)	0 (0.0)	1 (0.6)	
Prior exposure to GO (%)	No	107 (99.1)	30 (100.0)	19 (100.0)	4 (100.0)	160 (99.4)	0.92
	Yes	1 (0.9)	0 (0.0)	0 (0.0)	0 (0.0)	1 (0.6)	
Prior exposure to INO (%)	No	106 (98.1)	30 (100.0)	19 (100.0)	4 (100.0)	159 (98.8)	0.8
	Yes	2 (1.9)	0 (0.0)	0 (0.0)	0 (0.0)	2 (1.2)	
UDCA for SOS/VOD prophylaxis (%)	No	5 (4.6)	0 (0.0)	1 (5.3)	0 (0.0)	6 (3.7)	0.64
	Yes	103 (95.4)	30 (100.0)	18 (94.7)	4 (100.0)	155 (96.3)	

*The “others” disease category includes T-cell prolymphocytic leukemia, chronic active Epstein-Barr virus infection, etc.

*SOS/VOD* sinusoidal obstruction syndrome/veno-occlusive disease, *AML* acute myeloid leukemia, *ALL/LBL* acute lymphoblastic leukemia/lymphoma, *MDS* myelodysplastic syndrome, *CML/MPN* chronic myeloid leukemia/myeloproliferative neoplasm, *ML* malignant lymphoma, *MM* multiple myeloma, *ATL* adult T-cell leukemia/lymphoma, *AA* aplastic anemia, *CR* complete remission, *PR* partial remission, *SD* stable disease, *PD* progressive disease, *ECOG PS* Eastern Cooperative Oncology Group performance status, *HCT-CI* hematopoietic cell transplant comorbidity index, *BM* bone marrow, *PBSC* peripheral blood stem cell, *CB* cord blood, *HLA* human leukocyte antigen, *MAC* myeloablative conditioning, *RIC* reduced-intensity conditioning, *BU* busulfan, *TBI* total body irradiation, *GVHD* graft-versus-host disease, *TAC* tacrolimus, *MMF* mycophenolate mofetil, *CyA* cyclosporine, *GO* gemtuzumab ozogamicin, *INO* inotuzumab ozogamicin, *UDCA* ursodeoxycholic acid.

### Evaluation of the SOS/VOD diagnosis upon applying the refined EBMT criteria 2023

**Fig. 1:**
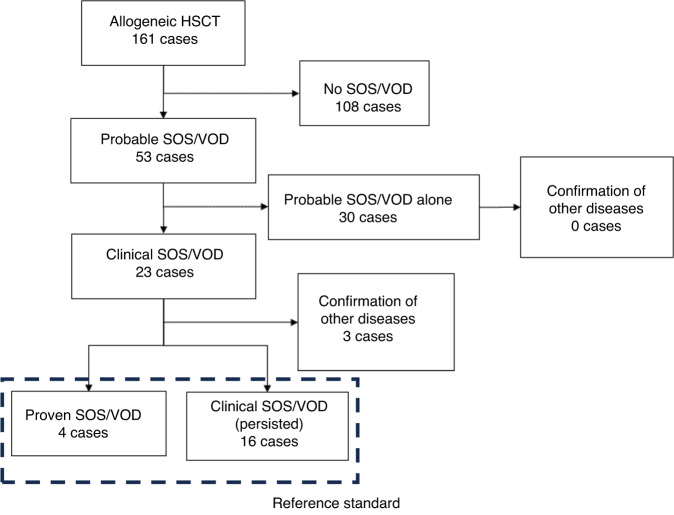
Patient flowchart. All “proven SOS/VOD” cases were histologically confirmed by autopsy. All three cases in which other diseases were confirmed were identified by means of a liver biopsy. Of 23 clinical SOS/VOD cases, five cases developed clinical SOS/VOD and probable SOS/VOD simultaneously. SOS/VOD sinusoidal obstruction syndrome/veno-occlusive disease, HSCT hematopoietic stem cell transplantation.

Upon applying the refined EBMT criteria 2023, we identified 53 probable SOS/VOD, 23 clinical SOS/VOD, and 4 proven SOS/VOD cases (Fig. 1). Of the 23 clinical SOS/VOD cases, 20 (10 classical SOS/VOD and 10 late onset SOS/VOD) were estimated as the reference standard. The remaining three cases were histologically confirmed as having other diseases (1 herpes simplex virus infection identified on autopsy, and 2 chronic graft-versus-host diseases identified on liver biopsy). We observed the development of the modified Seattle criteria for 54 cases. The sensitivity and specificity (95% confidence interval) of the modified Seattle criteria, probable SOS/VOD, and clinical SOS/VOD were 95.0%/75.2% (75.1–99.9/67.2–82.1), 100.0%/76.6% (76.2–100.0/68.7–83.3), and 100.0%/97.9% (76.2–100.0/93.9–99.6), respectively.

**Fig. 2:**
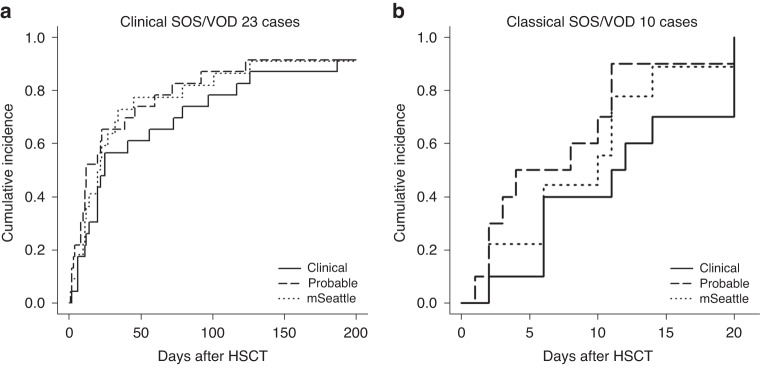
Cumulative incidence of each SOS/VOD. Cumulative incidence is described by considering death as a competing risk. **a** Cumulative incidence of each diagnostic criterion among clinical SOS/VOD cases. **b** Cumulative incidence of each diagnostic criterion among classical SOS/VOD cases. SOS/VOD sinusoidal obstruction syndrome/veno-occlusive disease, HSCT hematopoietic stem cell transplantation, mSeattle modified Seattle criteria.

Of the 23 clinical SOS/VOD cases, probable SOS/VOD was diagnosed a median of 5.0 days earlier (interquartile range [IQR]: 2.0–13.0 days, *P* < 0.001) than clinical SOS/VOD (Fig. S1A). Five cases developed clinical SOS/VOD and probable SOS/VOD simultaneously. Using the modified Seattle criteria, SOS/VOD was diagnosed a median of 1.0 days earlier (IQR: 0.0–10.5 days, *P* = 0.006) than clinical SOS/VOD. The difference in the number of diagnosed days between probable SOS/VOD and the modified Seattle criteria was insignificant (median: 0.0 days, IQR: 0.0–4.5 days, *P* = 0.209). In the classical SOS/VOD setting, probable SOS/VOD demonstrated a significant precedence over clinical SOS/VOD (median: 2.5 days, IQR: 0.5–5.75 days, *P* = 0.022), whereas the modified Seattle criteria did not (median: 0.0 days, IQR: 0–2 days, *P* = 0.181) (Fig. S1B). No significant difference between probable SOS/VOD and the modified Seattle criteria was observed in this setting (median: 0.0 days, IQR: 0–3 days, *P* = 0.201). Cumulative incidences of probable SOS/VOD, the modified Seattle criteria, and clinical SOS/VOD are shown in Fig. 2. At the time of probable SOS/VOD diagnosis, we identified 35 (66%) anicteric cases out of 53 probable SOS/VOD (Table 3). In addition, of the 23 cases eventually diagnosed with “clinical SOS/VOD”, 14 cases (61%) were initially anicteric at the time of their “probable SOS/VOD” diagnosis.

**Table 3:** SOS/VOD symptoms at the time of probable SOS/VOD diagnosis.

	*N*	Bilirubin ≥2 mg/dL	Painful Hepatomagaly	Weight gain	Ascites	Ultrasound finding
Probable SOS/VOD	53	18 (34%)	11 (21%)	40 (75%)	45 (85%)	3 (6%)
Clinical SOS/VOD	23	9 (39%)	6 (26%)	19 (83%)	19 (83%)	2 (9%)

*SOS/VOD* sinusoidal obstruction syndrome/veno-occlusive disease.

### Clinical impact of the refined EBMT criteria 2023

**Fig. 3:**
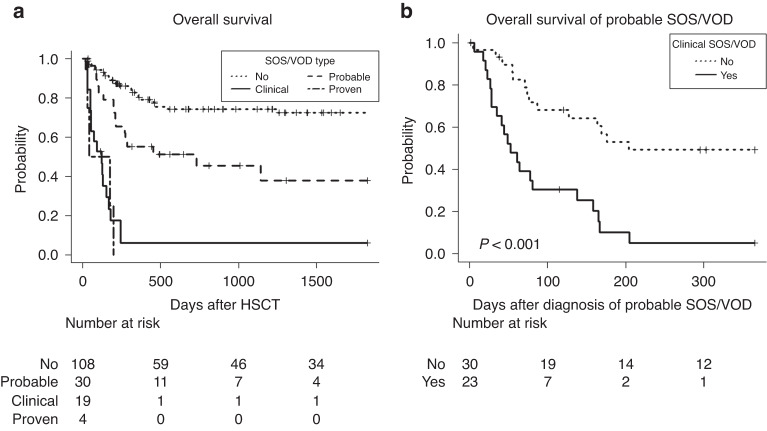
Association of SOS/VOD diagnosis with the overall survival. **a** Overall survival of the total cases, grouped by the maximally advanced diagnostic category of SOS/VOD. **b** Overall survival of probable SOS/VOD cases, grouped by the subsequent transition to clinical SOS/VOD. SOS/VOD sinusoidal obstruction syndrome/veno-occlusive disease, HSCT hematopoietic stem cell transplantation.

Overall survival is shown in Fig. 3a, grouped according to the maximally advanced diagnostic category of SOS/VOD. The development of clinical SOS/VOD was associated with high mortality (100-day survival, 52.2%). In addition, the development of “probable SOS/VOD alone” (i.e., without the development of clinical SOS/VOD) was associated with an inferior survival proportion than that of non-SOS/VOD cases (100-day survival, 86.2% vs. 94.3%). Post-hoc tests revealed the significance of survival proportions among the non-SOS/VOD, probable SOS/VOD, and clinical SOS/VOD groups (*P* < 0.001, with the exception *P* = 0.012 for non-SOS/VOD vs. probable SOS/VOD). Of the cases that developed probable SOS/VOD, the subsequent transition to clinical SOS/VOD was related to a significant deterioration in prognosis after probable SOS/VOD diagnosis (*P* < 0.001) (Fig. 3b). Elevated bilirubin levels at the time of probable SOS/VOD diagnosis were associated with an inferior prognosis, despite no subsequent transition to clinical SOS/VOD (Fig. S2).

### Evaluation of the SOS/VOD severity grading

**Fig. 4:**
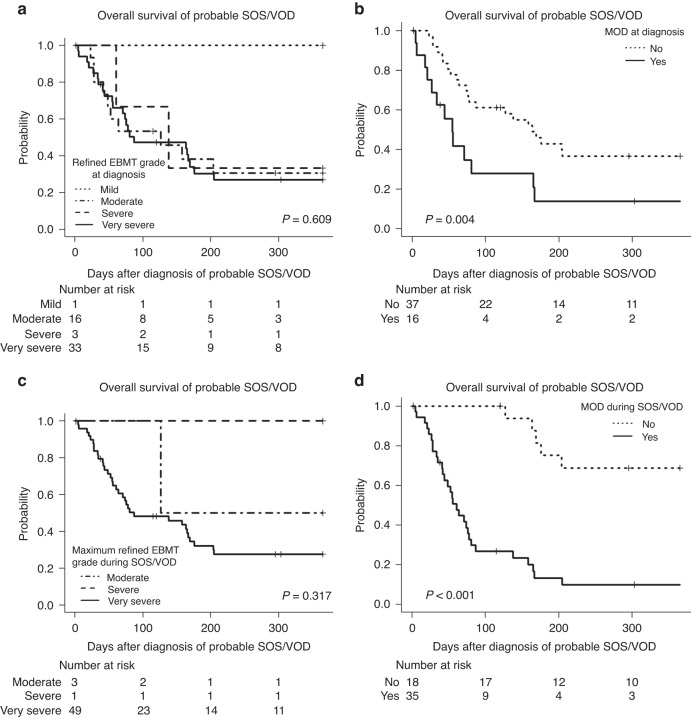
Association of SOS/VOD severity with the overall survival. Overall survival of probable SOS/VOD cases, grouped by (**a**) severity grade in the refined EBMT criteria 2023 and (**b**) MOD estimated by the SOFA score, at the diagnosis of probable SOS/VOD. Using the maximum grade during the entire clinical course after the diagnosis of probable SOS/VOD, the overall survival of probable SOS/VOD was grouped as (**c**) and (**d**). SOS/VOD sinusoidal obstruction syndrome/veno-occlusive disease, EBMT European Society for Blood and Marrow Transplantation, MOD multiple organ dysfunction.

All probable SOS/VOD cases were graded as shown in Table 4. The prognosis after probable SOS/VOD diagnosis is shown in Fig. 4. The presence of MOD, estimated using the SOFA score, could predict the prognosis significantly (Fig. 4b). In addition, the development of MOD during the entire clinical course after probable SOS/VOD diagnosis suggested that it could predict the prognosis more distinctly than that at diagnosis (Fig. 4d). Focusing on probable SOS/VOD alone, we obtained similar results that using the SOFA score improved the ability to predict the prognosis (Fig. S3). Of the probable SOS/VOD cases, we observed 11 relapse-related deaths out of 37 deaths. The estimation using TRM demonstrated a similar tendency (Fig. S4). We performed an identical evaluation focusing on clinical SOS/VOD (Table S3 and Fig. S5). No specific results for efficacy of the severity grading were obtained in the clinical SOS/VOD setting. In our cohort, we could not observe apparent effects of defibrotide. Finally, we investigated the factors associated with the transition from probable to clinical SOS/VOD. Eventually, no significant relationship was observed between the severity of SOS/VOD at probable SOS/VOD diagnosis and the subsequent transition to clinical SOS/VOD (Fig. S6).

**Table 4:** Severity of probable SOS/VOD cases.

		Probable SOS/VOD alone	Total probable SOS/VOD
	*N*	30	53
Refined EBMT criteria 2023 grade	Very severe at diagnosis	20	33
	Others at diagnosis	10	20
	Very severe in entire clinical course	26	49
	Others in entire clinical course	4	4
MOD estimated by SOFA	MOD (+) at diagnosis	8	16
	MOD (−) at diagnosis	22	37
	MOD (+) in entire clinical course	13	35
	MOD (−) in entire clinical course	17	18

*SOS/VOD* sinusoidal obstruction syndrome/veno-occlusive disease, *EBMT* European Society for Blood and Marrow Transplantation, *MOD* multiple organ dysfunction, *SOFA* sequential organ failure assessment.

## Discussion

Using the new “probable” diagnostic category, we could diagnose SOS/VOD significantly earlier than that using the conventional “clinical” category. The precedence of probable SOS/VOD may partially result from the dispensation of elevated bilirubin, which is considered to occur at a relatively later SOS/VOD phase [12]. Actually, using the probable SOS/VOD criteria, more than half of the clinical SOS/VOD cases were diagnosed at a phase with normal bilirubin levels in this study. While we did not observe a significant difference in the number of diagnosed days compared with that using the modified Seattle criteria, the newly induction of probable SOS/VOD may enable the earlier initiation of SOS/VOD treatment. Because the immediate initiation of SOS/VOD treatment is associated with improved clinical outcomes [6–8], it would potentially overcome the poor prognosis of SOS/VOD. Moreover, the development of “probable SOS/VOD alone” was associated with the deterioration of overall survival. Although some misdiagnosis or overlaps such as a severe infection supposedly exist particularly in patients with “probable SOS/VOD alone”, this result suggests new treatable entity in clinical practice. In pediatrics, anicteric SOS/VOD is often observed, which is the major reason for adopting the different SOS/VOD diagnostic criteria in pediatrics [22]. In contrast, anicteric SOS/VOD has been considered rare in adults [10, 23]. However, some reports have demonstrated that anicteric SOS/VOD can be seen even in adults to some extent, particularly in late onset SOS/VOD setting [24–26]. The new “probable SOS/VOD” category will facilitate understanding the substance of anicteric SOS/VOD in adults, which can never be detected as conventional “clinical SOS/VOD”. Furthermore, the category of “probable SOS/VOD” seemed to be valuable for clinical assessment in icteric probable SOS/VOD patients as well as in those with anicteric probable SOS/VOD, as shown in Fig. S2 because icteric probable SOS/VOD cases in this study showed a poor prognosis even without the subsequent progression to clinical SOS/VOD. Consequently, the identification of the newly proposed “probable SOS/VOD” is important in clinical practice.

Regarding SOS/VOD severity, newly incorporation of the SOFA score into the grading system appeared beneficial. MOD, as estimated using the SOFA score, could predict the prognosis of probable SOS/VOD. The SOFA score consists of more systemic parameters, such as cardiovascular and respiratory parameters, than the rest of estimations in the EBMT grading system. Systemic factors may influence the survival more strongly than SOS/VOD factor alone, particularly in probable SOS/VOD setting. Notably, MOD development during the entire clinical course after probable SOS/VOD diagnosis could predict the prognosis more distinctly. This result suggests that the estimation of SOS/VOD severity should be repeated after SOS/VOD diagnosis. Previously, some reports demonstrated that the conventional EBMT severity grading could predict the prognosis of clinical SOS/VOD [3, 20, 26]. It is not known sufficiently whether these results are similarly applicable to the refined EBMT criteria 2023, because this grading system is not originally intended to evaluate probable SOS/VOD. However, the newly incorporation of the SOFA score, which shows the efficacy to predict the prognosis in this study, is expected to improve the efficacy of severity grading additionally. Further investigations with a large sample size and improvements of the severity grading system are continually warranted.

Our results generated some issues. The adaptation of probable SOS/VOD may overdiagnosis, leading to initiation of redundant treatment, particularly in patients with probable SOS/VOD alone. “Probable SOS/VOD” was established and promoted by EBMT to facilitate earlier diagnosis and treatment, resulting in a relatively lower diagnostic accuracy when compared to clinical SOS/VOD. Defibrotide is widely used as a medication for SOS/VOD [27], whereas it can induce rare but potentially critical side effects, such as bleeding or hypotension [26]. Therefore, the application of defibrotide treatment should be considered carefully. In this study, the apparent effect of defibrotide was not observed, presumably due to the very small number of administered cases. However, considering that probable SOS/VOD alone cases with MOD and/or elevated bilirubin levels demonstrated an inferior prognosis in this study, defibrotide administration for such patients might be clinically valuable. Further studies are required to elucidate the efficacy of defibrotide in probable SOS/VOD and to identify groups for whom defibrotide treatment is effective. We did not identify specific factors associated with the transition from probable to clinical SOS/VOD in this study. No significant relationship was observed between SOS/VOD severity at probable SOS/VOD diagnosis and the subsequent transition to clinical SOS/VOD. It was challenging to elicit a valid answer for this unexpected result. However, the small sample size was considered as one of the reasons for it. It is crucial to differentiate SOS/VOD from other diseases that lead to endothelial injury syndromes and to determine whether probable SOS/VOD will progress to clinical SOS/VOD. Additional research is necessary to address this issue. From another perspective, even patients with non-severe probable SOS/VOD should be examined carefully for immediate intervention to prevent the deterioration of their general condition. Due to the recent introduction of probable SOS/VOD as a disease category, clinical decision-making for each case was based on the diagnosis of clinical SOS/VOD in its entirety in this study. Consequently, the effectiveness of making a clinical decision in the context of probable SOS/VOD remains unclear. Additional issues include discriminating SOS/VOD from other diseases. In this study, significant benefits of liver biopsy in the point of detecting SOS/VOD were not observed. However, a liver biopsy revealed other diseases in some clinical SOS/VOD cases, suggesting that it is an important examination for differentiating between various endothelial injury syndromes. Notably, the new concept of idiopathic portal hypertension-related refractory ascites has been recently reported, which is similar but histologically negative for SOS/VOD [28]. Consequently, efforts to confirm “proven SOS/VOD” will remain to be as important as an early diagnosis.

This study has some limitations. First, this is a small retrospective study conducted in a single institution. Second, not all cases were histologically confirmed to have SOS/VOD. Third, elastography [29, 30] was not performed at our institution; thus, its efficacy and contribution, compared with ultrasound remain unknown. Despite these limitations, this study is valuable in point of the first report indicating the utility of the refined EBMT criteria 2023.

In conclusion, the refined EBMT criteria 2023 demonstrated the utility of SOS/VOD diagnosis and severity grading, particularly for early detection. Adaptation of these new criteria would lead to more advanced clinical assessments of SOS/VOD and the improvement of clinical outcomes. Further investigations and improvements in these criteria are warranted.

### Supplementary information

Supplemental Figures and Tables

## Data Availability

Data supporting the findings of this study shall be made available from the corresponding author upon a reasonable request.
