# Supplementary material for: Utility of the refined EBMT diagnostic and severity criteria 2023 for sinusoidal obstruction syndrome/veno-occlusive disease
Source: Bone Marrow Transplant. 2024 Jan 29;59(4):518–25. doi: 10.1038/s41409-024-02215-4 (PMC10994840; doi:10.1038/s41409-024-02215-4)
Supplement: Supplementary file 1 — Supplemental Figures and Tables [file 41409_2024_2215_MOESM1_ESM.docx]

**Supplemental Figures**


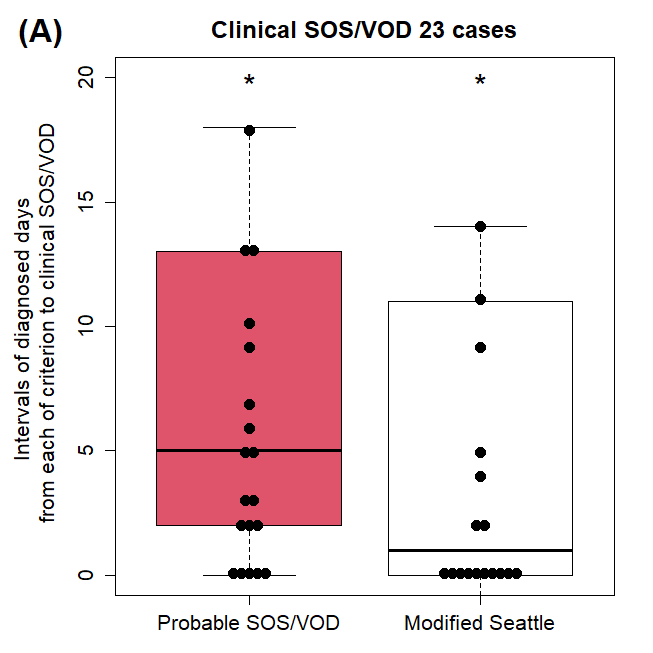

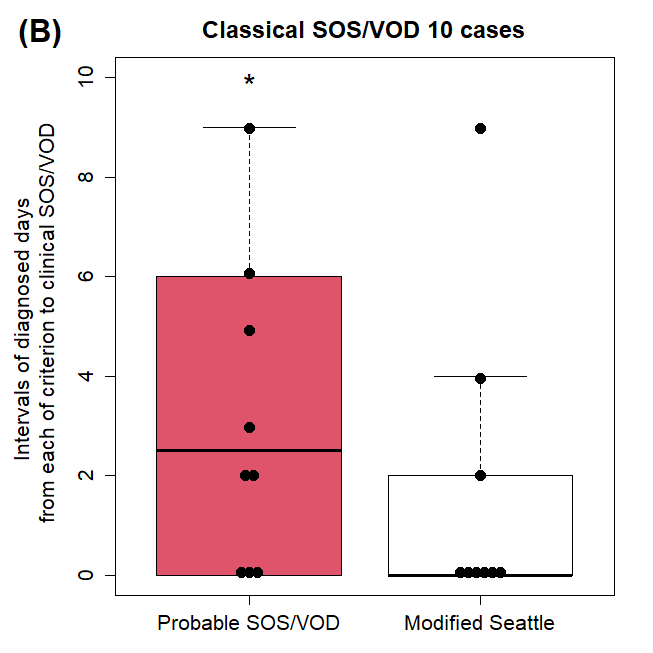


**Figure S1. Evaluation of the precedence of probable SOS/VOD**

Diagnostic precedence of each criterion to clinical SOS/VOD was plotted and described. * *P* < 0.05

Abbreviation: SOS/VOD, sinusoidal obstruction syndrome/veno-occlusive disease.


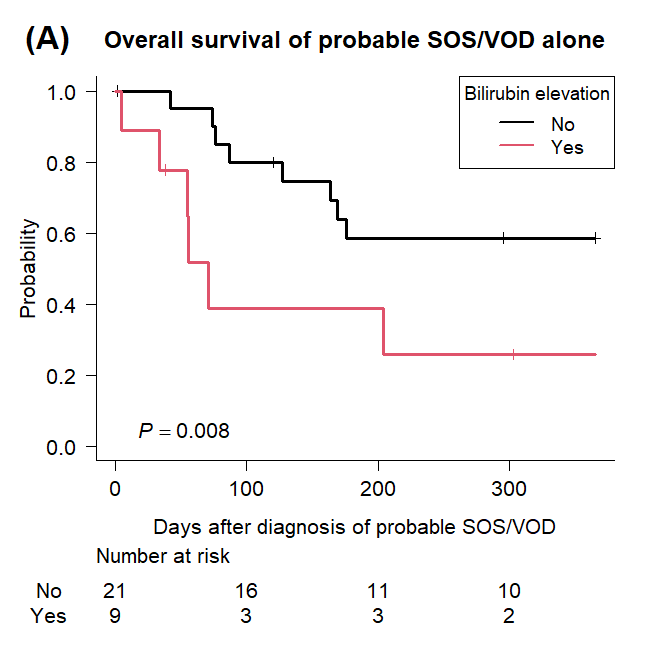

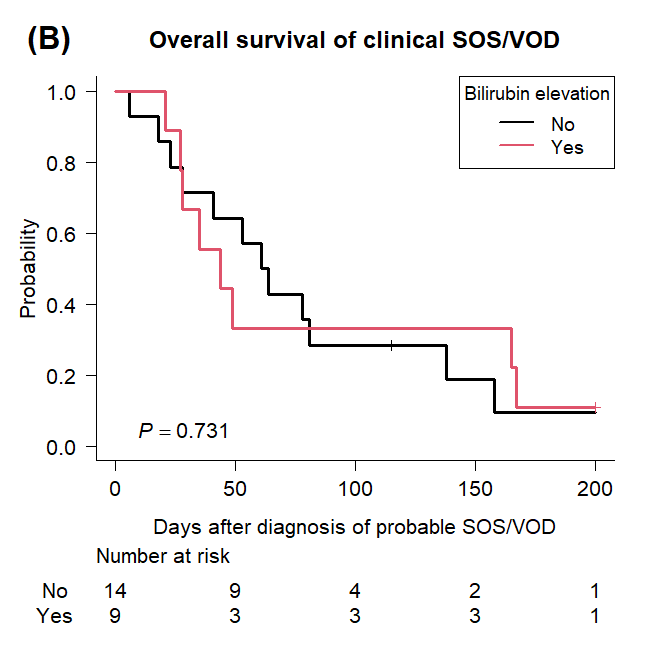


**Figure S2. Overall survival grouped by the presence of elevated bilirubin at the time of probable SOS/VOD diagnosis**

Prognosis after probable SOS/VOD diagnosis among (A) probable SOS/VOD alone and (B) subsequent clinical SOS/VOD transition cases, grouped according to the presence of elevated bilirubin at the time of probable SOS/VOD diagnosis.

Abbreviation: SOS/VOD, sinusoidal obstruction syndrome/veno-occlusive disease.


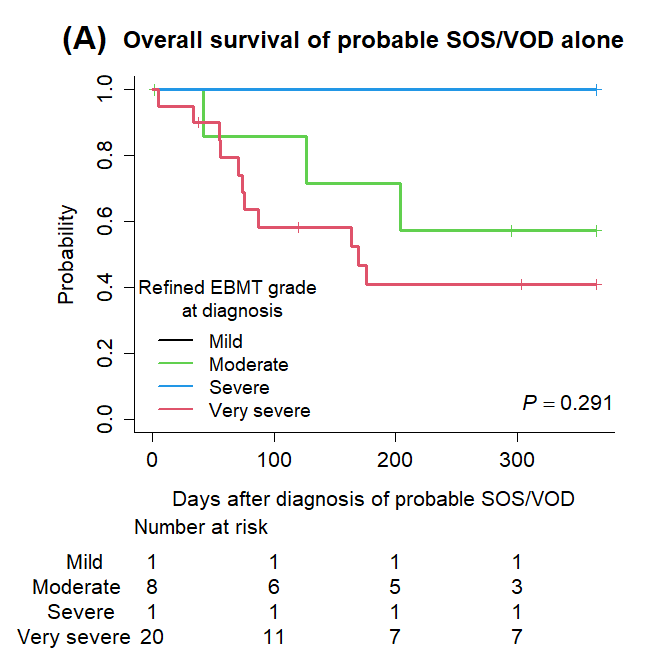

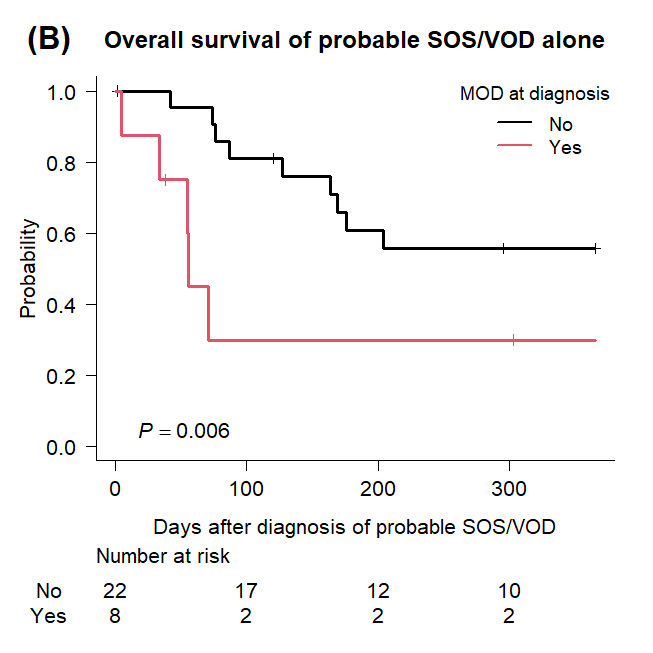

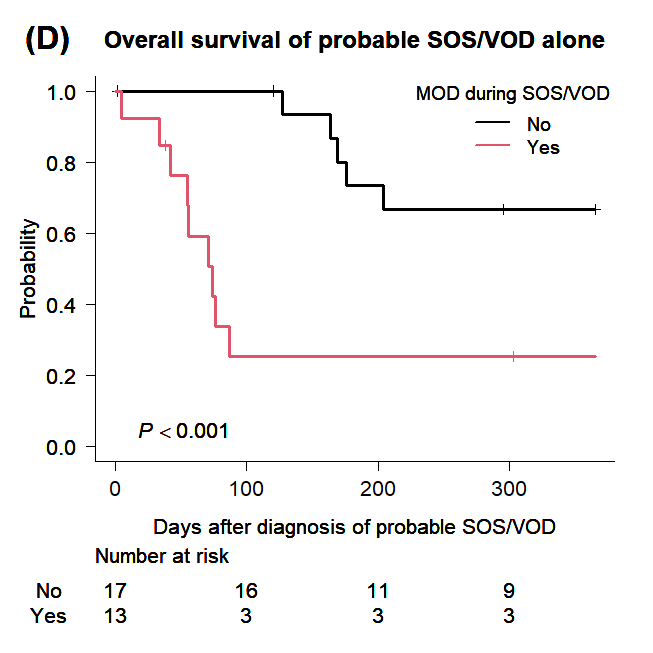

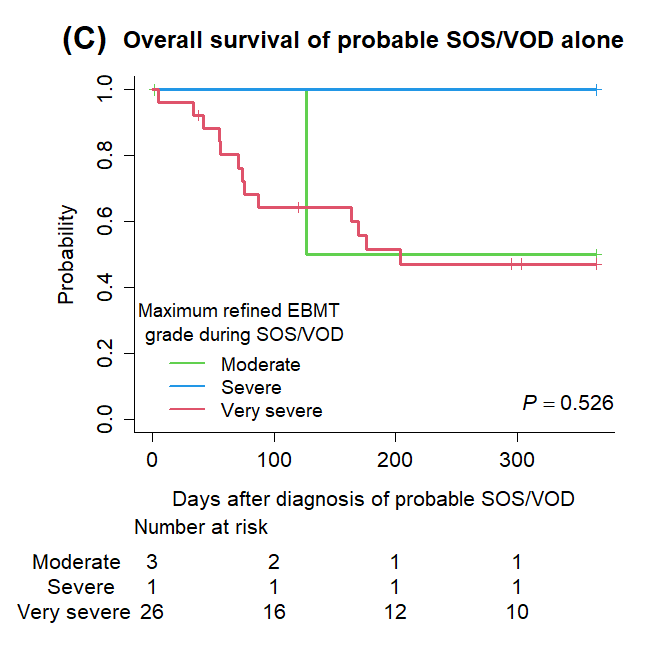


**Figure S3. Association of the SOS/VOD severity with overall survival in probable SOS/VOD alone**

Overall survival of probable SOS/VOD alone cases, grouped by (A) severity grade in the refined EBMT criteria 2023 and (B) MOD estimated by the SOFA score, at the diagnosis of probable SOS/VOD. Using the maximum grade during the entire clinical course after the diagnosis of probable SOS/VOD, the overall survival of probable SOS/VOD alone was grouped as (C) and (D).

Abbreviations: SOS/VOD, sinusoidal obstruction syndrome/veno-occlusive disease; EBMT, European Society for Blood and Marrow Transplantation; MOD, multiple organ dysfunction.


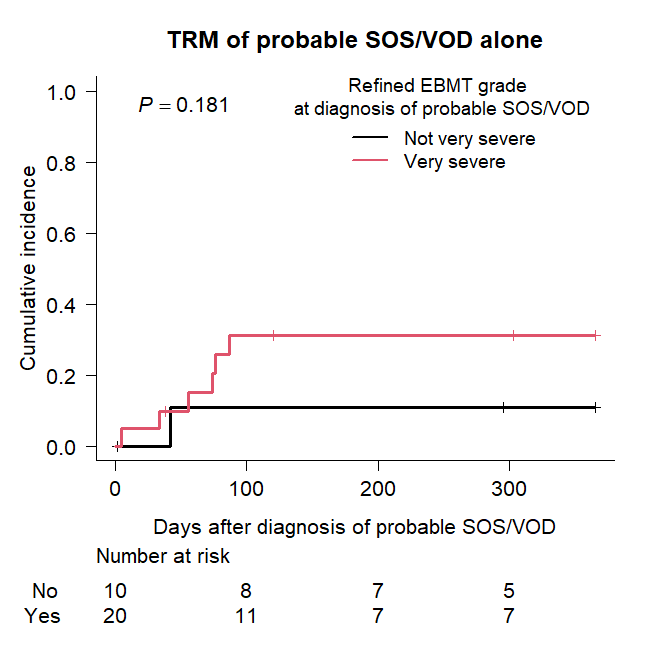

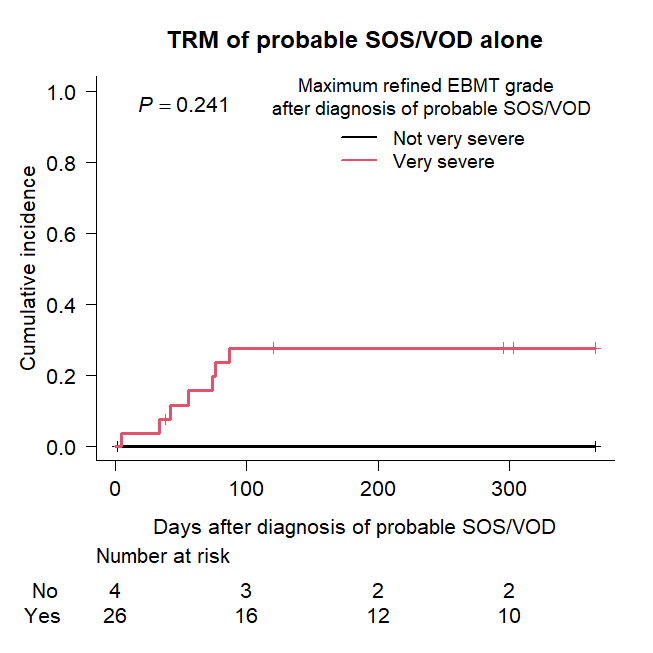


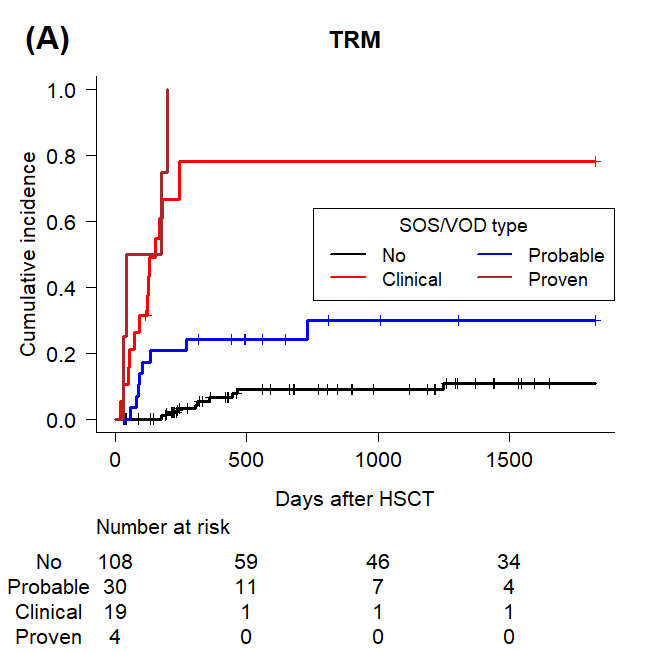

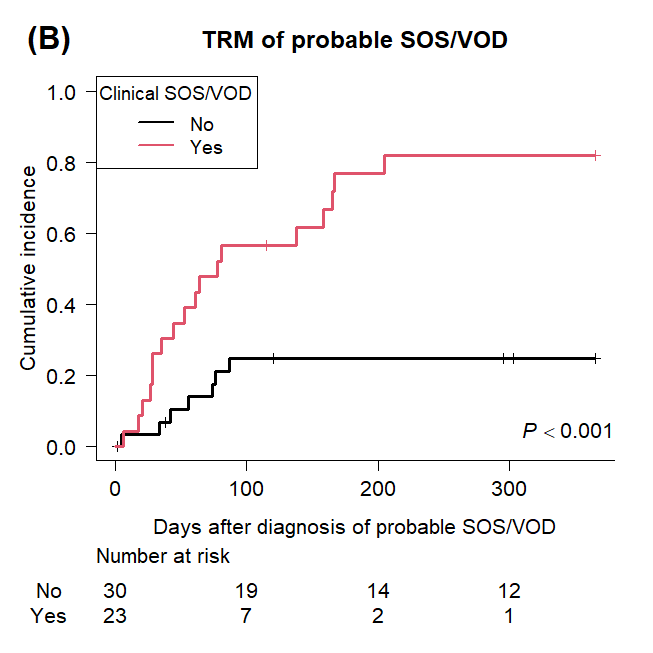

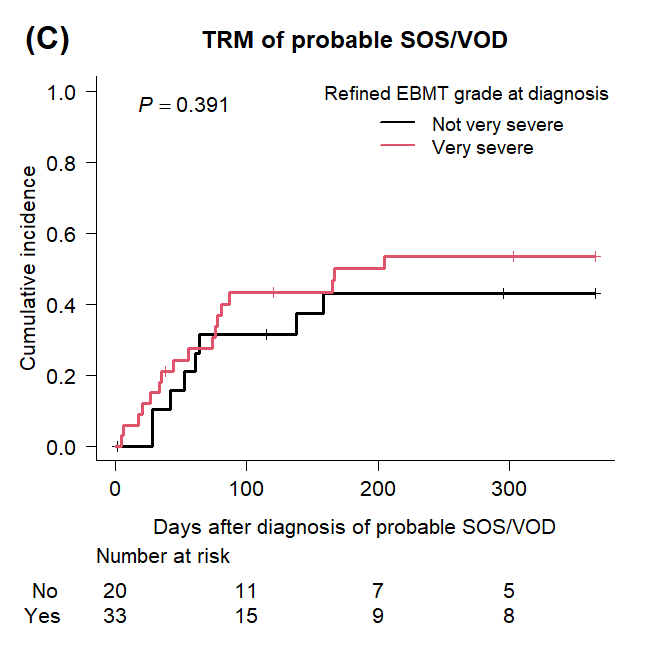

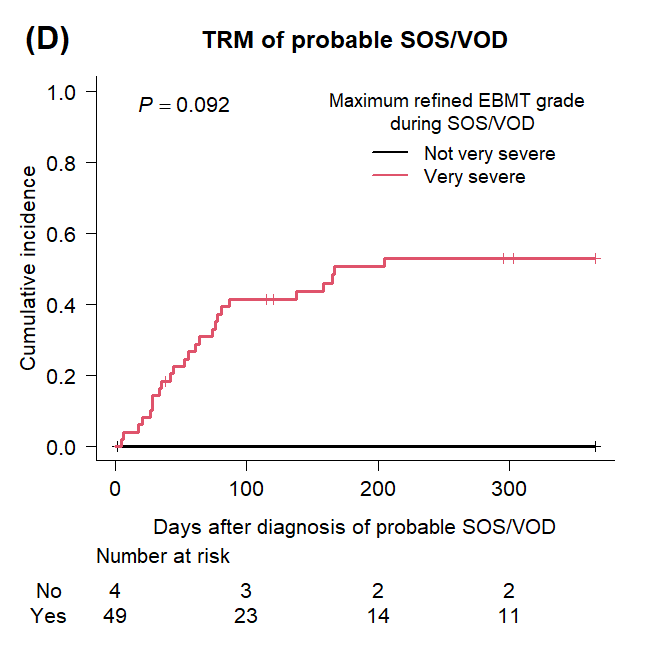

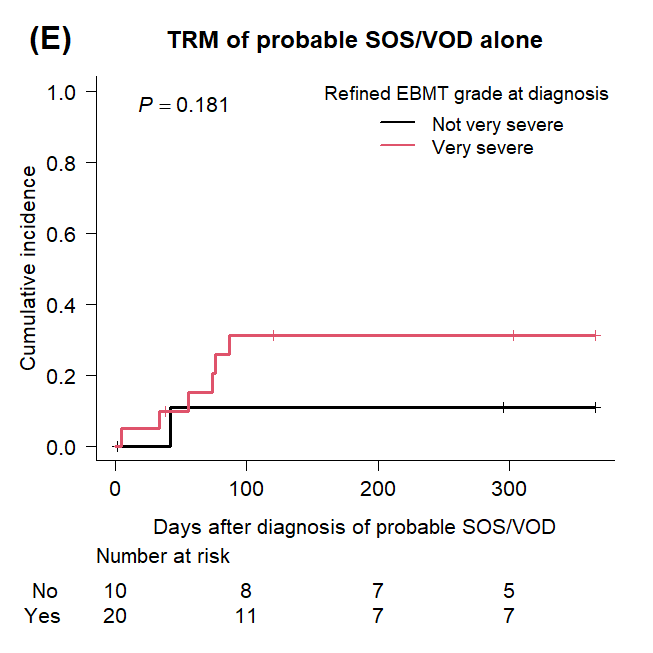

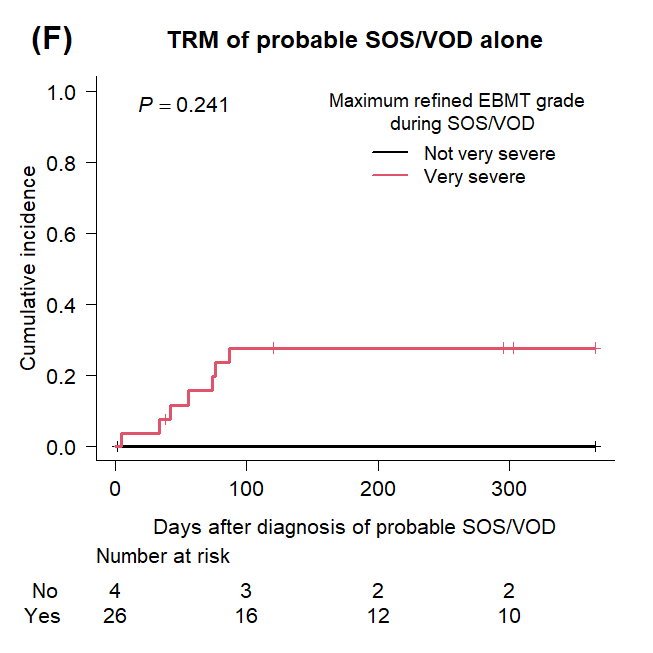


**Figure S4. Estimations of the prognosis using transplantation-related mortality**

Transplantation-related mortality was described using the cumulative incidence method, considering relapse-related death as a competing risk.

Abbreviations: TRM, Transplantation-related mortality; SOS/VOD, sinusoidal obstruction syndrome/veno-occlusive disease; HSCT, hematopoietic stem cell transplantation; EBMT, European Society for Blood and Marrow Transplantation.


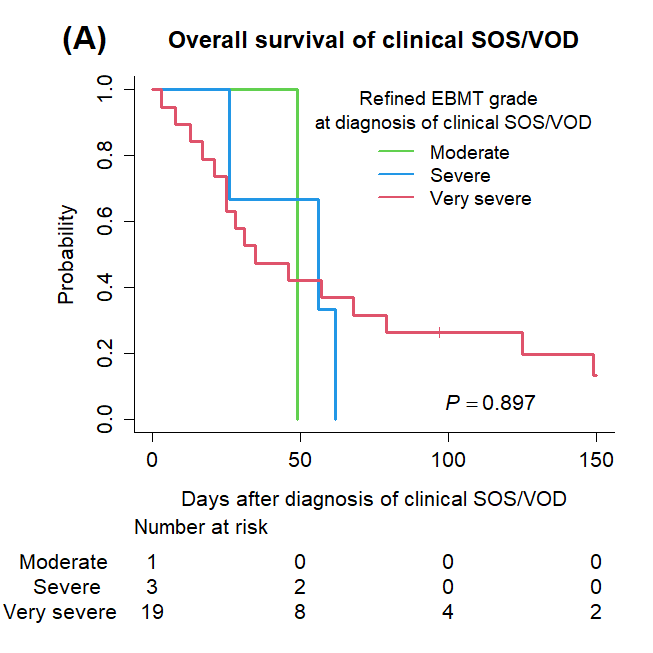

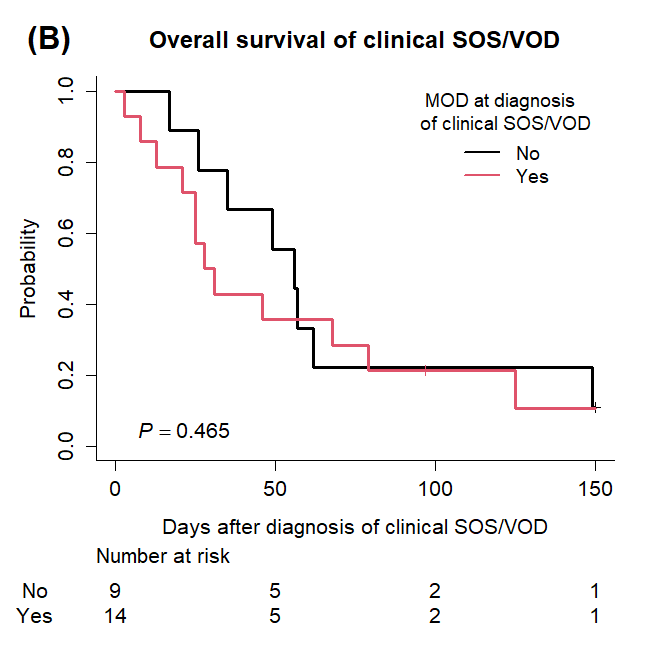

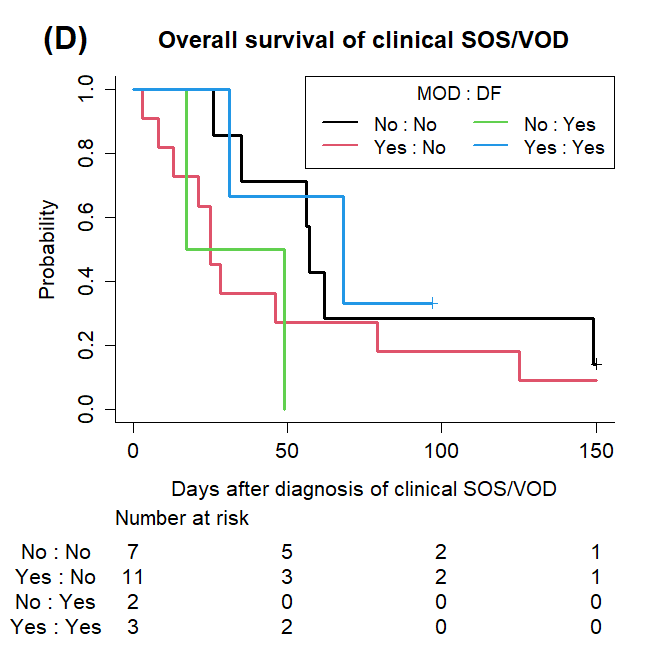

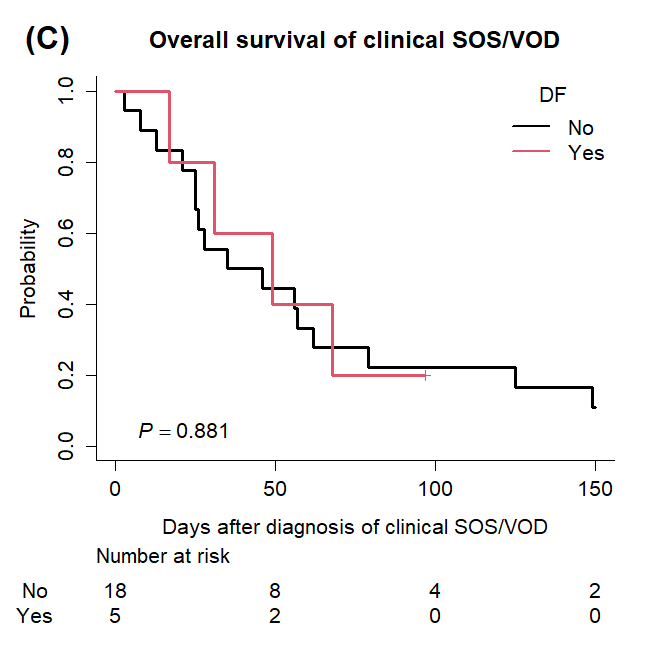


**Figure S5. Association of various factors with the overall survival in clinical SOS/VOD**

The overall survival of 23 clinical SOS/VOD cases, grouped by (A) severity grade in the refined EBMT criteria 2023 and (B) MOD estimated by the SOFA score, at the diagnosis of clinical SOS/VOD. The use of defibrotide grouped the overall survival of clinical SOS/VOD as (C) and (D).

Abbreviations: SOS/VOD, sinusoidal obstruction syndrome/veno-occlusive disease; EBMT, European Society for Blood and Marrow Transplantation; MOD, multiple organ dysfunction; DF, defibrotide.


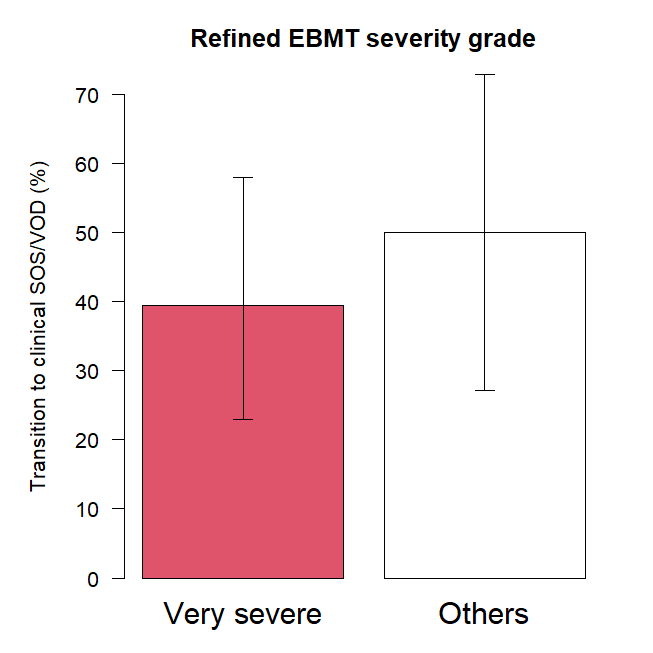

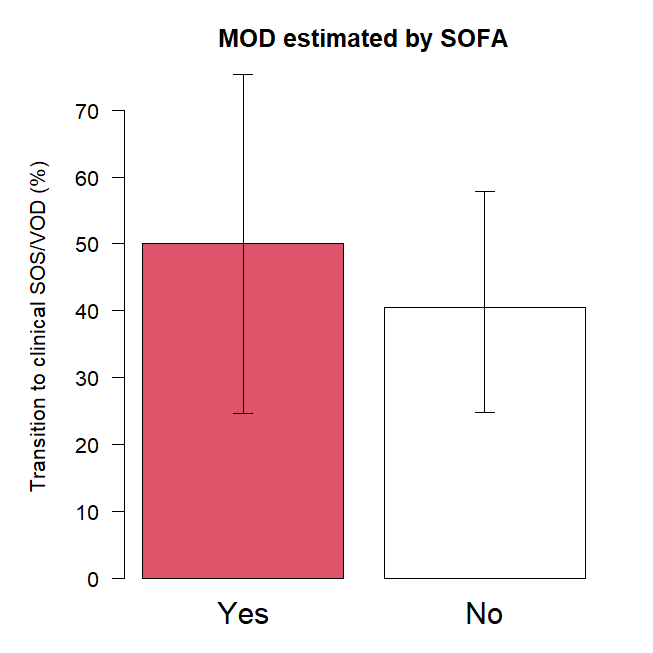


**Figure S6. Association of SOS/VOD severity with the subsequent transition to clinical SOS/VOD, among probable SOS/VOD cases**

Proportions of transition to clinical SOS/VOD, grouped by the SOS/VOD severity at the diagnosis of probable SOS/VOD. The 95% confidence intervals of each proportion are also described.

Abbreviations: SOS/VOD, sinusoidal obstruction syndrome/veno-occlusive disease; EBMT, European Society for Blood and Marrow Transplantation; MOD, multiple organ dysfunction; SOFA, Sequential Organ Failure Assessment.

**Supplemental Tables**

|  | Mild* | Moderate* | Severe | Very severe – MOD** |
| --- | --- | --- | --- | --- |
| Time since clinical symptoms of SOS/VOD | >7 days | 5–7days | <4 days | Any time |
| Bilirubin (mg/dL) | >2.0 and <3.0 | >3.0 and <5.0 | >5.0 and <8.0 | >8.0 |
| Bilirubin kinetic |  |  | Doubling within 48h |  |
| Transaminases | <2 x normal | >2 and <5 x normal | >5 and <8 x normal | >8 x normal |
| Weight increase |  |  | >5% and <10% | >10% |
| Serum creatinine | Baseline at transplant | >1 and <1.5 x baseline at transplant | >1.5 and <2 x baseline at transplant | >2 x baseline at transplant |

**Table S1. Severity grading in the refined EBMT criteria 2023**

* In case of presence of 2 or more risk factors for SOS/VOD, patients should be classified in the upper grade.

** Patients with MOD estimated by the SOFA score must be classified as ‘very severe.’

Abbreviations: SOS/VOD, sinusoidal obstruction syndrome/veno-occlusive disease; EBMT, European Society for Blood and Marrow Transplantation; MOD, multiple organ dysfunction; SOFA, Sequential Organ Failure Assessment.

|  | 0 | 1 | 2 | 3 | 4 |
| --- | --- | --- | --- | --- | --- |
| PaO2/FiO2 (mmHg) | Normal | >300 and <400 | >200 and <300 | >100 and <200 with respiratory support | <100 with respiratory support |
| Platelets (x 10^3^/μL) | Normal | >100 and <150 | >50 and <100 | >20 and <50 | <20 |
| Bilirubin (mg/dL) | Normal | >1.2 and <2.0 | >2.0 and <6.0 | >6.0 and <12.0 | >12.0 |
| Hypotension | Normal | MAP < 70mmHg | Dopamin <5 or Dobutamine any dose | Dopamin >5 and <15 or Epinephrine <0.1 or Norepinephrine <0.1 | Dopamin >15 or Epinephrine >0.1 or Norepinephrine >0.1 |
| GCS | Normal | 13–14 | 10–12 | 6–9 | <5 |
| Serum creatinine (mg/dL) or Urine output | Normal | >1.2 and <2.0 | >2.0 and <3.5 | >3.5 and <5.0 or >200 mL/day and <500mL/day | >5.0 or  <200 mL/day |

**Table S2. The SOFA score**

Abbreviations: SOFA, Sequential Organ Failure Assessment; MAP, mean arterial pressure; GCS, Glasgow Coma Scale.

|  |  | Transition to clinical SOS/VOD (+) | Transition to clinical SOS/VOD (-) |
| --- | --- | --- | --- |
|  | N | 23 | 30 |
| Refined EBMT | Very severe | 13 | 20 |
| criteria 2023 grade | Others | 10 | 10 |
| MOD estimated | MOD (+) | 8 | 8 |
| by SOFA | MOD (-) | 15 | 22 |

**Table S3. Association of severity at the diagnosis of probable SOS/VOD with the subsequent transition to clinical SOS/VOD**

Abbreviations: SOS/VOD, sinusoidal obstruction syndrome/veno-occlusive disease; EBMT, European Society for Blood and Marrow Transplantation; MOD, multiple organ dysfunction; SOFA, Sequential Organ Failure Assessment.
